# Metal organic framework-coated gold nanorod as an on-demand drug delivery platform for chemo-photothermal cancer therapy

**DOI:** 10.1186/s12951-021-00961-x

**Published:** 2021-07-19

**Authors:** Junfeng Huang, Zhourui Xu, Yihang jiang, Wing-cheung Law, Biqin Dong, Xierong Zeng, Mingze Ma, Gaixia Xu, Jizhao Zou, Chengbin Yang

**Affiliations:** 1 Shenzhen Key Laboratory of Special Functional Materials & Shenzhen Engineering Laboratory for Advance Technology of Ceramics, College of Materials Science and Engineering, Shenzhen University, Shenzhen, 518060 China; 2 Key Laboratory of Optoelectronic Devices and Systems of Ministry of Education and Guangdong Province, College of Physics and Optoelectronic Engineering, Shenzhen University, Shenzhen, 518060 China; 3 Guangdong Key Laboratory for Biomedical Measurements and Ultrasound Imaging, School of Biomedical Engineering, Shenzhen University Health Science Center, Shenzhen, 518060 China; 4 Department of Industrial and Systems Engineering, The Hong Kong Polytechnic University, Hong Kong, 999077 China; 5 Guangdong Provincial Key Laboratory of Durability for Marine Civil Engineering, College of Civil and Transportation Engineering, Shenzhen University, Shenzhen, 518060 China

**Keywords:** Metal organic framework, Gold nanorod, Photothermal therapy, Drug delivery, Biocompatible

## Abstract

Chemo-photothermal therapy based on nanoparticles has emerged as a promising strategy for cancer treatment. However, its therapeutic efficacy and application potential are largely subjected to the uncontrollability and biotoxicity of functional nanoplatforms. Herein, a novel biocompatible and biodegradable metal organic framework (MOF), which was constructed by growing crystalline zeolitic imidazolate framework-8 on gold nanoroad (Au@ZIF-8), was designed and fabricated for efficient drug loading and controlled release. Owing to the large surface area and guest-matching pore size of ZIF-8, doxorubicin (DOX) was successfully loaded into the Au@ZIF-8 with a high drug loading efficiency of ~ 37%. Under NIR light or weakly acidic environment, the ZIF-8 layer was quickly degraded, which resulted in an on-demand drug release in tumour site. More importantly, under the irradiation of near infrared (NIR) laser, highly efficient cancer treatment was achieved in both in vitro cell experiment and in vivo tumour-bearing nude mice experiment due to the synergistic effect of photothermal (PTT) therapy and chemotherapy. In addition, the in vivo study revealed the good biocompatibility of Au@ZIF-8. This work robustly suggested that Au@ZIF-8 could be further explored as a drug delivery system for chemo-photothermal synergistic therapy.

**Supplementary Information:**

The online version contains supplementary material available at 10.1186/s12951-021-00961-x.

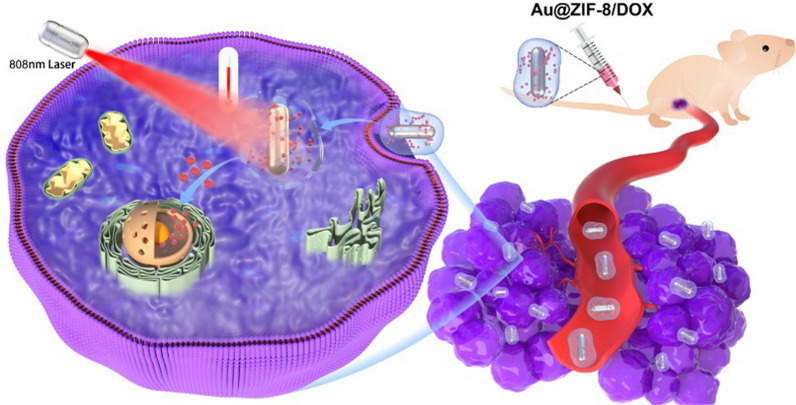

## Background

Cancer, as the most dreadful disease in the world, was responsible for nearly one-fifth of human death [1]. Owing to the great efforts contributed to the biomedical field in the past few decades, several cancer therapy approaches, such as chemotherapy [2], surgery [3], and radiotherapy [4], have been developed and used in clinical settings. As a major cancer therapeutic approach, the chemotherapy still faces many challenges in clinical practice, such as limited therapeutic efficacy, poor patient compliance and severe toxic-side effects [5]. In recent years, thanks to the encouraging progress of nanotechnology-based combined therapy [6], which integrated different components, such as drugs, targeting molecules, biodegradable polymers, into a single nanoplatform, has emerged as a potential solution to overcome the aforementioned hurdles. The synergism of various components not only contributed to a remarkable super-additive therapeutic effect, but also significantly reduced the adverse effects [7–9]. Among diverse types of combinations, chemo-photothermal therapy has attracted special attentions for four major reasons: 1) hyperthermia could potentially sensitize tumor to chemo-drugs, 2) hyperthermia could enhance the cellular uptakes of drug carriers, 3) the localized drug release can be triggered by the photothermal effects [10], and 4) chemo-drug could efficiently interrupt the cell metabolism and soften the heat shock response upon hyperthermia [11]. All these features have enabled us to obtain satisfactory outcomes of cancer therapy with reduced side effects. Nevertheless, efficiency of chemo-photothermal therapy depends on the design and fabrication of nanoplatforms. Currently, core–shell nanostructures have become a gold standard due to the high flexibility to control the size, morphology, and individual function [12]. Hence, this work is focused on selecting safe and efficient functional components to fabricate a multifunctional nanoplatform for chemo-photothermal therapy.

With the continuous efforts in photothermal applications, various types of photothermal agents have been explored, including copper chalcogenide nanocrystals [13], nanocarbons [14], gold-based nanostructures [10, 15], black phosphorous[16], and organic dyes [17]. Among them, gold-based nanomaterials have more excellent performance for clinical translation owing to its promising photothermal responses and bio-inertness [18–20]. Up to now, four clinical trials (ClinicalTrials.gov identifier: NCT00848042, NCT01679470, NCT02680535 and NCT04240639) of photothermal therapy based on gold nanostructures have been endorsed by the National Institute of Health Clinical Center (U.S.A), suggesting that the great application potential of gold-based nanostructures. Currently, anisotropic gold nanorod (AuNR) with tunable optical properties has been demonstrated to be an ideal component for constructing core–shell nanoplatform for tumour therapy [21, 22]. When AuNR was used as a photothermal core, on-demand controllable drug release from the shell reservoir could be triggered by light excitation [23]. However, limited choices have been given for shell materials due to their low drug loading capability, toxic synthetic approaches, time-consuming synthesis, and high tendency in causing aggregation [24, 25]. Therefore, in order to achieve the full potential of chemo-photothermal therapy, it is critical to choose biocompatible, biodegradable, and reliable shell materials.

Recently, metal–organic frameworks (MOFs), which composed of metal nodes and organic linkers, have emerged as promising materials for biomedical applications, such as drug delivery [26, 27], catalysis [28], molecular imaging [29], and biosensing [30], due to their exceptional large surface area and tunable inner cavity. Among them, zeolitic imidazolate framework-8 (ZIF-8) with low-toxic Zn^2+^ and 2-methylimidazole (2-MIN) as the basic units, was recognized as a biocompatible and acid-responsive drug carrier [31]. In addition, the fabrication process requires no toxic additives with facile synthetic approach, which ensure the safety use and reliability of ZIF-8 nanostructures. Inspired by its unique properties, we envisaged a great opportunity to exploit the full potential of chemo-photothermal therapy by combining AuNR and ZIF-8 shell materials.

In this work, a multifunctional nanoplatform composed of AuNR, ZIF-8 and doxorubicin (DOX) were successfully designed and synthesized for chemo-photothermal synergistic therapy. Specifically, a core–shell formulation of Au@ZIF-8 was prepared by growing ZIF-8 material onto individual AuNR with the guidance of Polyvinylpyrrolidone (PVP) polymer. Ascribed to the remarkable surface area and guest-matching pore size of ZIF-8, an exceptional drug loading efficiency of ~ 37% were achieved in Au@ZIF-8/DOX formulation. Furthermore, the materials characteristics results demonstrated that the weak acidic condition and photothermal effect promoted degradation of ZIF-8 shell layer, which resulted in an on-demand controlled drug release. The Au@ZIF-8 showed a high photothermal effect upon NIR irradiation, and the generated heat not only directly killed cancer cells but also synergistically promoted the DOX release, which exhibited better performance for inhibiting cell viability than free DOX. Furthermore, in vivo therapeutic results confirmed that the synergistic chemo-photothermal by Au@ZIF-8/DOX + NIR achieved much higher treatment efficacy than photothermal therapy by Au@ZIF-8 + NIR or chemotherapy by DOX only and even resulted in complete tumor elimination without obvious adverse effect. This work provides a novel design of Au@ZIF-8 nanoplatform for chemo- and photothermal therapy, which can lead to a synergistic therapeutic effect and hold great promise for future clinical translation.

## Materials and methods

### Materials

Cetyltrimethylammonium bromide (CTAB) was purchased from Sigma. Tetra-chloroauric acid (HAuCl_4_), ascorbic acid (99.0%), silver nitrate (AgNO_3_), 2-methylimidazolate (2-MIM), Zinc nitrate hexahydrate, monosodium phosphate (NaH_2_PO_4_), sodium hydrogen phosphate (Na_2_HPO_4_), sodium borohydride (NaBH_4_), polyvinyl pyrrolidone (PVP) were purchased from Aldrich. Doxorubicin Hydrocholoride (DOX) was purchased from Aladdin. 1,2-distearoyl-sn-glycero-3-phosphoethanolamine-N-[methoxy(polyethylene glycol)-2000] (ammonium salt) (18:0 PEG2000PE) was purchased from Avanti. All the chemicals were used as received without further purification.

### The synthesis and characterization of nanocomplexes

Transmission electronic microscopy (HR-TEM, JEOL-F200; TEM, HTI-7700) was used to observe the morphology and size of Au@ZIF-8. Absorbance spectra of all the nanoparticle samples were recorded by using UV–Vis-NIR spectrophotometer (Tianjin Tuopu Instrument, TP-720). The crystal information of Au@ZIF-8 samples were measured by an X-ray Diffractometer (Rigaku Smartlab, JP). The photothermal performances of the samples were evaluated by an 808 nm laser (Changchun Leishi Photo-Electric Technology) and FLIR A300 infrared thermal imaging camera (FLIR Systems).

### The synthesis of AuNRs and Au@ZIF-8

Gold nanorods (AuNRs) were synthesized in a seed-mediated growth method. For the seed solution, 250 μL of 0.01 M HAuCl_4_ was added to 10 mL of 0.1 M CTAB with rapid stirring. Ice-cold 600 μL of 0.01 M NaBH_4_ solution was prepared and quickly dissolved into the mixture solution. The colour changed to dark brown, indicating the synthesis of gold seeds. The seed solution kept undisturbed for 2 h at 30 ℃ before use. For growth solution, 40 mL of 4 mM CTAB was mixed with 2 mL of 0.01 M HAuCl_4_, following with adding of 400 µL 0.01 M AgNO_3_ aqueous solution. The pH value of mixture was adjusted into 1 by addition of 1 M HCl. Then 320 µL of 0.10 M ascorbic acid was added and the colour of the mixture became transparent after mild stirring. 400 µl seed solution was added into growth solution and the mixture kept undisturbed in 4 h at 30 ℃ for the growth of gold nanorods. The brown precipitations were collected with 12,000 rpm centrifugation and dried for use in the next step.

Prior to grow ZIF-8 shell, a ligand exchange of CTAB-stabilized AuNR with PVP polymer is necessary. Briefly, 5 g PVP was dissolved into 100 mL DMF to form a homogeneous PVP/DMF solution. Next, the CTAB-stabilized AuNR was firstly precipitated by centrifugation and then resuspended in PVP/DMF solution with vigorous stirring for 2 h. The AuNR@PVP was then collected by centrifugation. To grow ZIF-8 shell on AuNR, 2 mL of 22 mM 2-MIM and 2 mL of 13 mM Zn(NO_3_)_2_ were added into 2 mL AuNR@PVP solution under vigorous stirring (2 h) at room temperature. Then, Au@ZIF-8 was collected by centrifugation and washed with methanol twice. To endow water dispersity and biocompatibility on Au@ZIF-8, PEG2000PE was firstly added into the Au@ZIF-8 methanol suspension with 2:1 weight ratio, and then PEG2000PE-stabilized Au@ZIF-8 were added into water under ultrasonication. Finally, the water dispersible Au@ZIF-8 were obtained by centrifugation.

### The evaluation of photothermal effect

The photothermal response of Au@ZIF-8 was studied on a simple setup. Briefly, a plastic tube containing 200 uL of sample dispersion with different concentrations were immobilized on an optical holder. 808 nm laser irradiation with 1 W·cm^−2^ power density was reflected by a 45°mirror to shed on nanorod samples in plastic tube. A thermal camera with an accuracy of 0.1 °C was operated in a perpendicular way to laser path for temperature detection. The heat conversion efficiency was calculated by using Eq. 1–4.

1 $$\eta {\text{ = }}\frac{{{\text{hS(}}{T_{max}} - {T_{ssur}}) - {Q_{dis}}}}{{{\text{I(1 - }}{{10}^{ - {A_{808}}}})}}$$

2 $${\tau }_{S}=\frac{{m}_{D}{C}_{D}}{hS}$$

3 $$\mathrm{t}=-{\tau }_{S}ln\theta$$

4 $$\uptheta =\frac{T-{T}_{surr}}{{T}_{max}-{T}_{surr}}$$

where "h" is the heat transfer coefficient, "S" is the surface area of the container, the value of "hS" can be obtained according to the cooling curve. T_max_ is the steady-state temperature of photothermal agents while T_ssur_ is the surrounding temperature. Q_dis_ represents the energy absorbed by the container and solvent. "I" is the power of incident laser power (1 W· cm ^−2^) while A808 is the absorbance of photothermal agents at 808 nm.

Drug loading and controlled release of AuNR@ZIF-8 by pH response or NIR trigger DOX was used as anti-cancer drug for detailed drug loading and release tests. DOX was dissolved into DMF to form DOX solutions (3.7 mM) and then mixed with 1 mg·mL^–1^ AuNR@ZIF-8 DMF solution overnight, the mixture was denoted as the Au@ZIF-8/DOX. Various ratio of DOX and Au@ZIF-8 were performed to seek the ideal drug loading content and efficiency. The drug loading content and efficiency of DOX (DLC_DOX_ & DLE_DOX_) were obtained according to the below equations respectively:

5 $${DLC}_{DOX}=\frac{{W}_{loaded}}{{W}_{total}}=\frac{{W}_{original}{-W}_{residual}}{{W}_{loaded}{+W}_{nanoparticle}}$$

6 $${DLE}_{DOX}=\frac{{W}_{original}{-W}_{residual}}{{W}_{original}}$$

where W_loaded_, W_total_ and W_nanoparticles_ corresponded to the weight of loaded DOX, the weight of total micelles and the weight of DOX carriers (nanoparticles) respectively; And W_original_ and W_residual_ corresponded to the weight of DOX before and after mixing with AuNR@ZIF-8 respectively. The residual weight of DOX was measured according to the standard curve for the DOX quantification. All data for DOX were adjusted by the standard curve in Additional file 1: Fig. S2. The in vitro drug release test was performed in PBS solutions. Equivalent Au@ZIF-8-DOX concentration (1 mg·mL^−1^ DOX) was used in different control group. There were four contrast groups in the tests: pH = 7.4 group without NIR irradiation, pH = 7.4 group with NIR irradiation, pH = 5.8 group without NIR irradiation and pH = 5.8 group under NIR irradiation. PBS solution was made up of NaH_2_PO_4_ and Na_2_HPO_4_ solution. During drug release tests, laser power, irradiation time and frequency were adjusted for optimal drug release. The amount of the released DOX was quantified by UV–Vis spectroscopy as the above-mentioned method.

### In vitro cytotoxicity of Au@ZIF-8

The cytotoxicity of Au@ZIF-8 was investigated by using HeLa cells and MCF-7 cells. The cells were seeded in 96-well plates at a density of 5000 cells/well and grown in 5% CO^2^ at 37 °C. Then, Au@ZIF-8 with diverse concentrations were supplemented into the medium and the cells were incubated for another 24 h. 10 μL of 3-[4,5-dimethylthiazol-2-yl]-2,5-diphenyltetrazolium bromide (MTT) solution was further added into each well and incubated for another 4 h. The supernatant in each well was discarded and supplemented with 150 μL of dimethyl sulfoxide (DMSO). The plate was examined using a microplate reader (Bio-tek, Epoch-2) at the wavelength of 490 nm to evaluate the cell viability. To evaluate the PTT efficacy of Au@ZIF-8, diverse concentrations of Au@ZIF-8 were firstly added into the medium of each well and incubated for 4 h. After replacement with fresh medium, the cell samples were exposed under 808 nm laser of 1.0 W·cm^−2^ for 7 min. After that, the cells were further cultured for 24 h. The MTT process was carried out as mentioned to assess the cell viability.

### Live and dead cell staining assay

To evaluate and visualize the PTT effect on Au@ZIF-8-treated cell samples, Live/Dead Cell Double Staining Kit was applied on cell samples after photothermal treatment. Briefly, 2 uL working solution of Calcein-AM and PI were added into each well of 6-well plate. After incubation of 15 min and replacement with fresh culture medium, the cell samples were imaged by fluorescent microscopy. Calcein-AM is a cell-permeable and non-fluorescent compound, however, becomes fluorescent once enters into metabolically active cells. PI is a fluorescent nucleic acid dye that can permeate only the damaged cell membrane.

### Hemolysis assay

The impact of the surface chemistry of Au@ZIF-8 on red blood cells (RBCs) of mice and the influence of the protein corona interaction on this process was investigated by the standard haemolysis assays. A volume of 4 mL of blood was added to the anticoagulant tube. The blood was mixed gently and centrifuged at 3,500 rpm for 15 min. The supernatant was removed, and the precipitated RBCs were washed three times by re-suspending them in a 1 × PBS solution (pH 7.4). The final working suspension utilized for the haemolysis assay consisted of 5% (v/v) of RCBs in a PBS solution. To evaluate the haemolytic effect, Au@ZIF-8 with diverse concentrations were incubated with RBCs (200 μL of a 5% suspension) for 2 h through a static method after gentle homogenization. The final volume of the haemolysis assay was set to be 1.0 mL. Then, the samples were centrifugated and 100 μL of supernatants were extracted for quantifying the haemoglobin by measuring the absorbance at 540 nm. De-ionized (DI) water and PBS solution were used as positive control and negative control, respectively. The percentage of haemolysis was calculated based on Eq. 7.

7 $$\mathrm{Hemolysis\, rate }\left(\mathrm{\%}\right)=\frac{{ABS}_{sample}-{ABS}_{neg-contl}}{{ABS}_{posit-contl}-{ABS}_{neg-contl}}\times 100\mathrm{\%}$$

### In Vivo photothermal therapy

The tumour-suppressive effects of Au@ZIF-8 with or without 808 nm laser irradiation were evaluated on nude mice (~ 20 g), which were purchased from Guangdong Medical Laboratory Animal Center. All animal experiments conform to the guidelines of the University Animal Care and Use Committee. The right axilla of each mouse was injected with MCF-7 cells subcutaneously to establish tumours. The tumours were allowed to grow for fourteen days to reach a size of around 200 mm^3^. Then, the MCF-7 tumour-bearing BALB/c mice were randomly divided into 8 groups (N = 4), which were intravenously injected with PBS, DOX, Au@ZIF-8, and DOX-loaded Au@ZIF-8, with or without laser irradiation. The injected dose of DOX solution (in PBS) was 3.6 mg·kg^−1^. The injected dose of Au@ZIF-8 suspension (in PBS) was 10 mg·kg^−1^ body weight in total. The photothermal treatment was performed on day 1. The body weight and tumour volumes were recorded every two days. The tumour volume was calculated using the following equation: Tumour Volume (V) = π × length × width^2^/8 × 4/3. Moreover, the tumour tissues in each group were harvested from mice 24 h after the first photothermal treatment. The tumour tissues were further fixed in paraformaldehyde and stained with hematoxylin and eosin (H&E) staining assay.

### In vivo biocompatibility assay

**Scheme 1.**
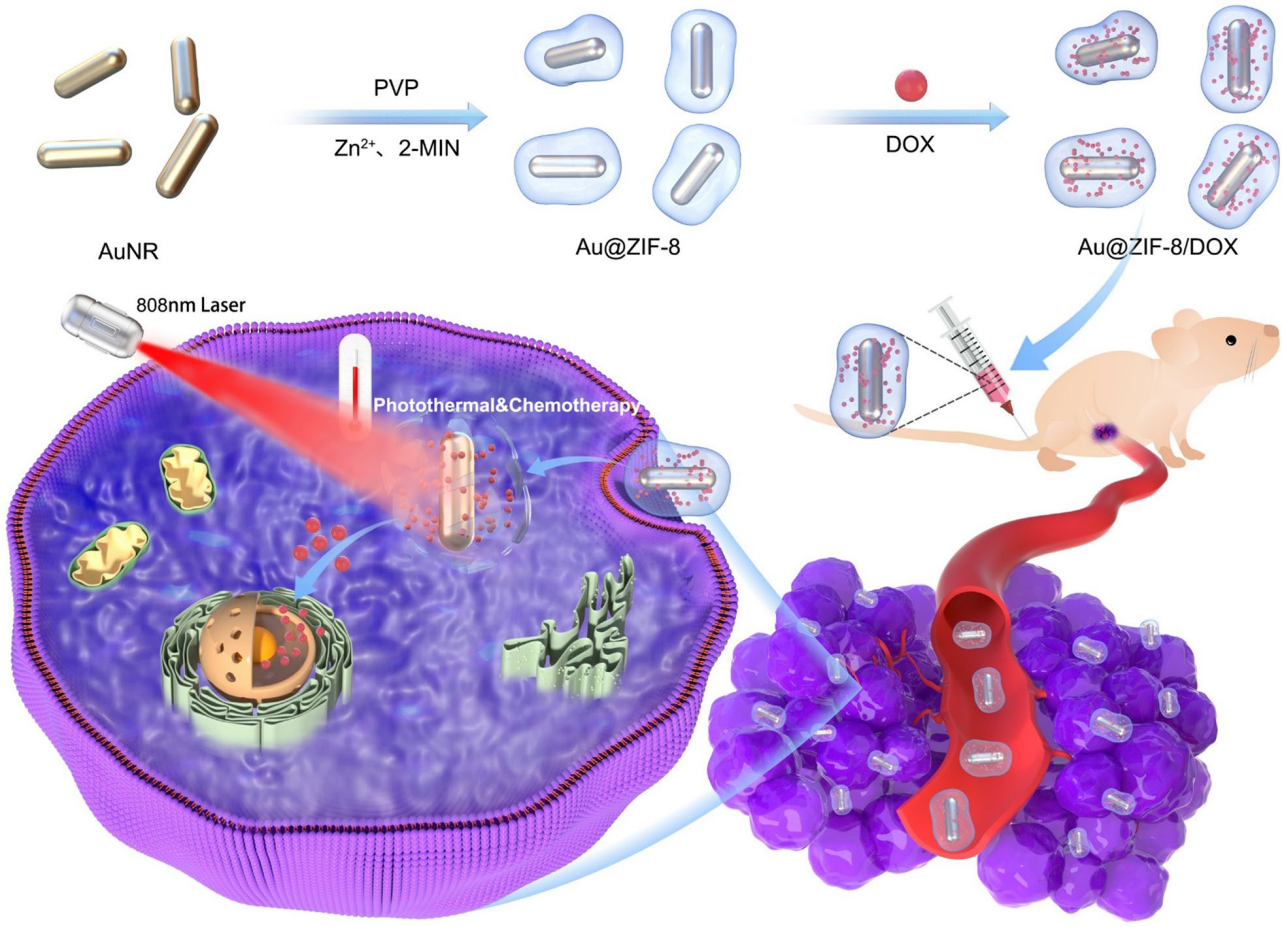
The schematic illustration of the synthetic procedure of Au@ZIF-8/DOX nanocomplexes for the chemo-photothermal synergistic cancer therapy in vivo

The in vivo toxicity of Au@ZIF-8 was carried out by using BALB/c mice as animal models. PBS was injected as the negative control. Au@ZIF-8 with three dose schemes (1, 10, and 20 mg·kg^−1^) were applied on BALB/c mice (n = 4). The body weight of mice in each group were recorded for every two days. On day 15, all mice were sacrificed. Their major organs (heart, liver, spleen, lung, and kidney) were retrieved to analyse the organic coefficient and perform H&E staining. Their blood samples were taken to characterize the blood routine test and blood biochemical test.

### Statistical analysis

Data were represented as means ± SD. The representative experimental data were indicated as mean ± standard deviation (SD). The statistical was examined by Student’s *t*-test when two groups were compared. Differences were considered statistically significant when *P-* values lower than 0.05.

## Results and discussion

### Fabrication and characterization of Au@ZIF-8 nanocomplex

**Fig. 1:**
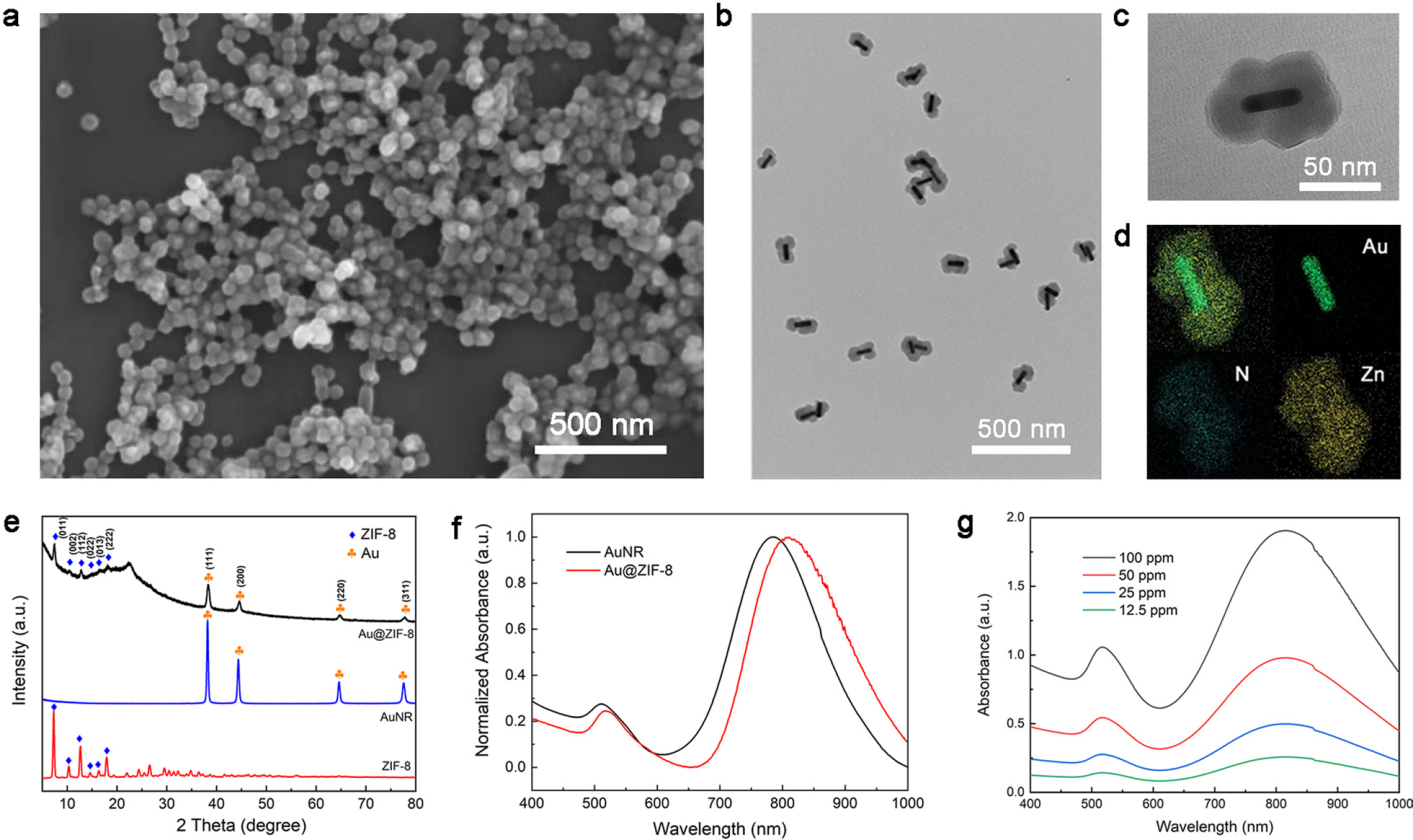
The characterization of Au@ZIF-8 nanocomplexes. **a** The morphology and distribution of Au@ZIF-8 indicated by SEM image. The inner structure, detailed diameter and composition are presented by **b** low magnified TEM image, **c** high magnified TEM image, and **d** EDX-elemental mapping. **e** The crystal information was examined byXRD patterns. **f** The absorption spectra of Au@ZIF-8 and AuNR were measured by spectrophotometer. **g **The absorption spectra of different concentrations of Au@ZIF-8

The fabrication process of drug loaded Au@ZIF-8 and its therapeutic mechanism are illustrated in Scheme 1. Firstly, AuNRs were prepared according to the classical seed-mediated method with cetyltrimethylammonium bromide (CTAB) as the capping agents [32]. By further exchanging the ligands with PVP polymer, crystallized ZIF-8 layer was gradually growing on AuNR by chelation reaction between Zn^2+^ and 2-MIN in methanol. Then, PEG2000PE was applied as capping agent to endow Au@ZIF-8 with water solubility and biocompatibility. Ascribed to the facile preparation process and enhanced water dispersity, highly monodispersed nanoparticles with round ellipsoid shape were obtained (Fig. 1a). This could be explained by the homogeneous-growing of shell layer. The inner structure of Au@ZIF-8 was examined by TEM microscopy. The core–shell conformation was clearly presented with AuNR as core and ZIF-8 as shell (Fig. 1b). As shown in Fig. 1c, the aspect ratio of AuNR core is about ~ 3.9 and thickness of MOF shell layer is about ~ 25 nm. The composition of Au@ZIF-8 was further confirmed by energy-dispersive X-ray (EDX) elemental mapping. Figure 1d shows a rod-shaped Au element map in the core and homogeneous distribution of nitrogen (N) and zinc (Zn) element in ellipsoid shape, which further confirmed the core–shell formulation of Au@ZIF-8. XRD spectrum also evidenced that ZIF-8 was successfully crystallized and capped around AuNR core. The signal pattern of Au@ZIF-8 well matches with face-centred cubic structure of gold and ZIF-8 (Fig. 1e), which as corresponding to the results implied by TEM images and EDX mapping. The absorption spectrum of Au@ZIF-8 was shown in Fig. 1f. Compared to AuNR, the absorption peak of Au@ZIF-8 slightly redshifted from 790 to 808 nm. This phenomenon could be explained by the difference of refractive index around AuNR, thereby altering the frequency of localized surface plasmon resonance [33]. In addition, the absorption spectra of Au@ZIF-8 with different concentrations was measured, in which a distinct absorption peak at ~ 800 nm was observed (Fig. 1g). Therefore, the photothermal effect of the prepared Au@ZIF-8 was set at 808 nm wavelength in the following experiments.

### Photothermal effect and on-demand drug release

**Fig. 2:**
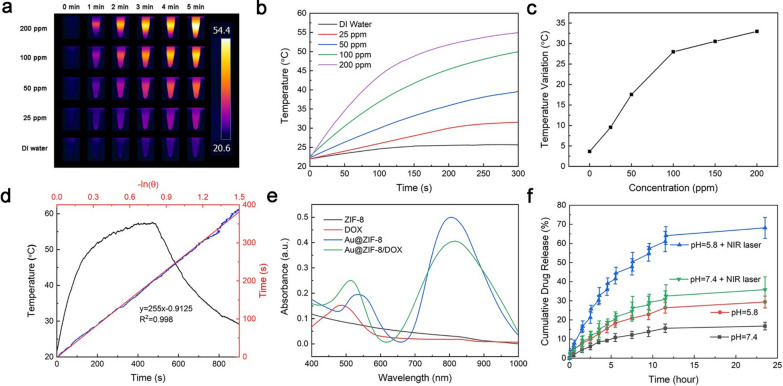
The photothermal responses and controlled drug release of Au@ZIF-8. **a** The temperature gradience of Au@ZIF-8 in diverse concentration under laser irradiation for 5 min. **b** The temperature elevation curve and **c** the final temperature of Au@ZIF-8 at different concentrations irradiated by 808 nm NIR laser for 5 min. **d** The photothermal and natural cooling curve of Au@ZIF-8. **e** The absorption spectra of different formulations**. f** The cumulative drug releasing curve of Au@ZIF-8/DOX under different conditions. (Laser: 808 nm, 1 W·cm^−2^)

The photothermal responses of Au@ZIF-8 were evaluated under the irradiation of 808 nm laser (1 W·cm^−2^). The result revealed that the temperature elevation of Au@ZIF-8 has positive correlation with the concentration of samples and the irradiation time (Fig. 2a, b). Typically, the temperature of Au@ZIF-8 suspension at high concentration of 200 ppm increased quickly, from 23 ℃ to 54 ℃, within 5 min and was finally stabilized at 54 ℃. However, the temperature variation of deionized (DI) water irradiated by NIR laser was only 2.5 °C, under the same experimental condition (Fig. 2b), indicating that the increase of temperature was due to the photothermal conversion of AuNRs. It is noted that the rate of increase of temperature was not linearly related to the concentration of Au@ZIF-8. The temperature of Au@ZIF-8 increased as ~ 0.25 ℃·ppm^−1^ below 100 ppm while dropped to 0.05 ℃·ppm^−1^ with higher concentration (Fig. 2c). The sensitive light-to-heat conversion was ascribed to the promising photothermal heating conversion efficiency (HCE) of AuNR. Based on Eqs. 1, 2, 3 and 4 in the experimental section and Fig. 2d, the HCE of Au@ZIF-8 was calculated to be 22%, which was comparable to AuNR alone and implies that ZIF-8 coating does not affect the photothermal effect of AuNR. Moreover, the heat energy producing from Au@ZIF-8 can be used to facilitate the rapid release of drug payloads in the pores of outer shell. Drug loading content and loading efficiency of DOX were assessed in aqueous solution (pH 7.4) at room temperature. As shown in Additional file 1: Fig. S1, the colour of mixed solution changed into mild purple after DOX loading. The detailed results were collected from absorbance spectra and calculated based on Eqs. 5 and 6 in experimental section. DOX standard curve was measured prior to ensure the accuracy of results (Additional file 1: Fig. S2). The drug loading content of Au@ZIF-8 was stable around 36% in different molar ratio and reached the highest at 37.32% with molar ratio of 3 (Additional file 1: Table S1). The drug loading efficiency showed a decreased trend when the molar ratio increased. The highest efficiency was 92.8% when the molar ratio was 1. Therefore, the ratio of Au@ZIF-8 and DOX in the coming tests were fixed as 1:1. It can be deduced that the excellent drug loading efficiency of Au@ZIF-8 is attributed to the high surface area after the encapsulation of MOF shell. The absorption spectra of different formulations (AuNR, ZIF-8, Au@ZIF-8, DOX and Au@ZIF-8/DOX) were shown in Fig. 2e. Generally, ZIF-8 demonstrated weak absorption between 400 and 1000 nm, suggesting that the influence of MOF on the optical absorption of AuNR is minimal. It may also provide additional explanation for the comparable HCE between AuNR and Au@ZIF-8. After loading DOX, the maximum absorption peak of Au@ZIF-8 mildly shifts from 528 to 512 nm.

Then, the drug release behavior was investigated in different pH environmental conditions (Fig. 2f). Obviously, the DOX release rate under acidic condition (pH 5.8) was much higher than that under neutral condition. Within 24 h, over 26% of DOX was released at pH 5.8, while only 13% of DOX was released for pH 7.4, which might be attributed to the degradation of MOF under acidic condition. TEM images clearly showed that the shell of Au@ZIF-8 underwent gradual decomposition under acid condition. Naked AuNRs were observed due to structural collapse and disappears of outside MOF shell. (Additional file 1: Fig. S3a). Moreover, the impact of temperature evaluation on the drug release was also evaluated. The results indicated that laser irradiation significantly enhanced DOX release. Upon the NIR irradiation, the release rate (68%) of DOX at acidic condition was 33% faster than that at pH 7.4, which confirms that the photothermal effect of Au@ZIF-8 can accelerate the drug release. The generated heat energy from photothermal effect further promoted the instability of ZIF-8 under acid condition (Additional file 1: Fig. S3b), thereby improving the release rate of DOX. The engineering of MOF shell structure onto AuNRs can also efficiently avoid the premature leakage of DOX under physiological conditions, reducing the adverse effect of chemotherapeutic drugs. Therefore, this photo and pH dual-responsive drug delivery manner of Au@ZIF-8/DOX was greatly desired for the controlled release of DOX at acidic tumour environment, thus improving its therapeutic effect.

### Intracellular internalization and in vitro chemo-photothermal therapy

**Fig. 3:**
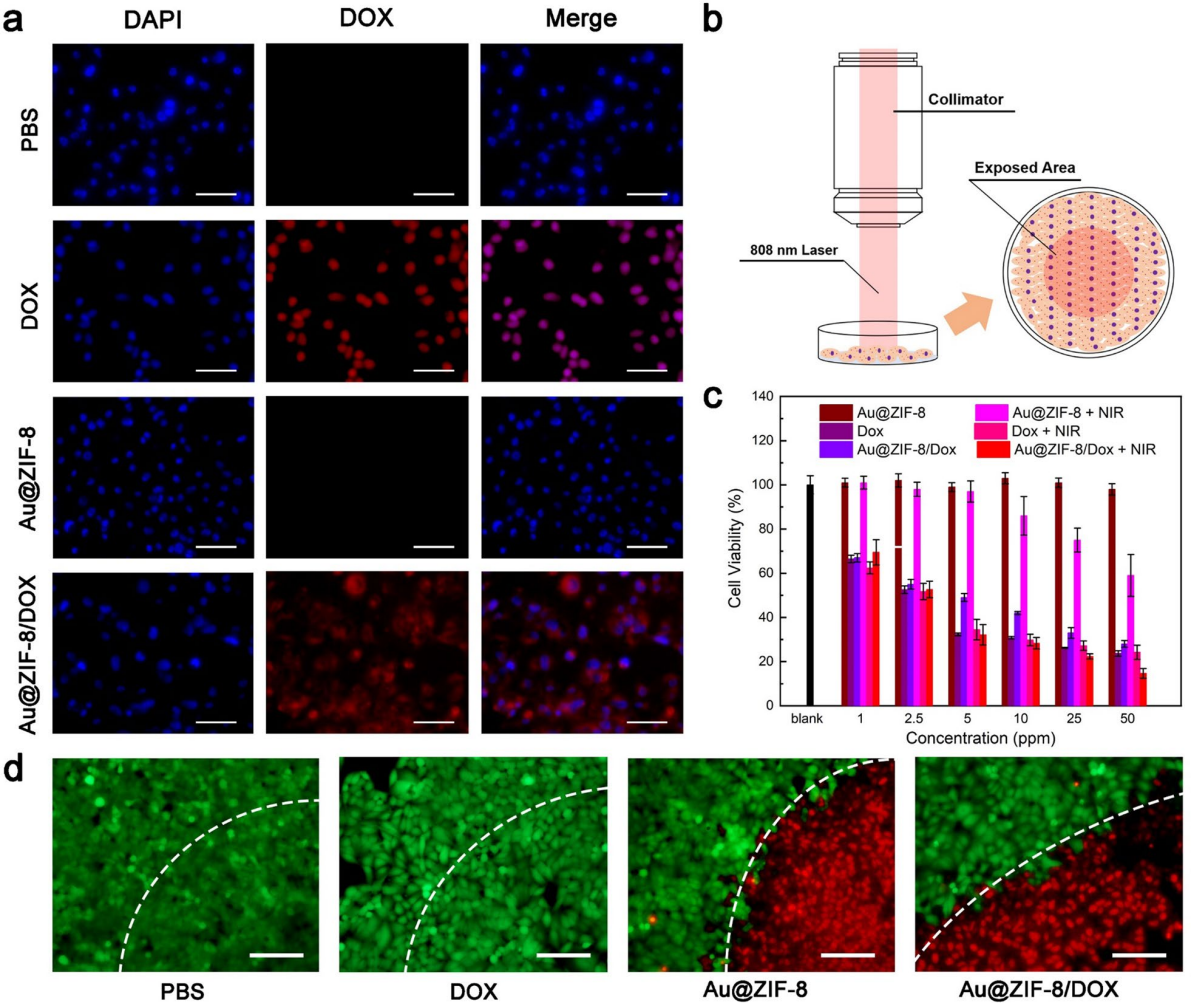
The in vitro chemo-photothermal therapy. **a** The fluorescent images of MCF-7 cells treated with PBS, DOX, Au@ZIF-8, and Au@ZIF-8/DOX without laser treatment and live/dead staining after laser treatment. **b** The schematic illustration of in vitro PTT. **c** The cell viability of MCF-7 cells treated with different formulations (PBS, DOX, Au@ZIF-8, and Au@ZIF-8/DOX) at various concentrations with or without 808 nm laser. Data was represented as mean ± SD (n = 4). **d** The corresponding fluorescent images of live-dead staining cells for different groups. Scar bar: 100 μm

The cellular uptake of Au@ZIF-8 was visualized under confocal laser scanning microscopy (CLSM). MCF-7 cells were incubated with DOX-loaded Au@ZIF-8 (Au@ZIF-8/DOX) for 4 h, followed by staining with LysoTracker green, which was a commercial fluorescent dye used for indicating the location of lysosomes. The red fluorescence of DOX and green fluorescence of LysoTracker green are well overlapped, implying that endocytosis process was the major route to internalize Au@ZIF-8 (Additional file 1: Fig. S4). Additionally, it was noted that the DOX fluorescence was majorly distributed outside the nucleus. After treated with PBS, DOX, Au@ZIF-8, and Au@ZIF-8/DOX, the MCF-7 cells were observed under CLSM (Fig. 3a). It can be seen that blue fluorescence which represents cell nucleus can be clearly visualized in all samples. The red fluorescence of DOX can only be observed in DOX- and Au@ZIF-8/DOX-treated cells. In addition, DOX-treated cells have a well overlapped fluorescence profile of red and blue while the red fluorescence in Au@ZIF-8/DOX treated-cells largely distributed around blue fluorescence. This phenomenon should attribute to the delayed DOX release behaviour of Au@ZIF-8 when compared with the free DOX. After the cell internalization, the photothermal effect of Au@ZIF-8 on anti-cancer effect was investigated, MCF-7 cells incubated with different formulations were irradiated by 808 nm laser (Fig. 3b). Next, the therapeutic efficacy of Au@ZIF-8 were further studied by MTT assay on MCF-7 cells. Briefly, PBS, DOX, Au@ZIF-8, and Au@ZIF-8/DOX were incubated respectively with MCF-7 cells. The concentrations of Au@ZIF-8 used in this experiment were set as (1, 2.5, 5, 10, 25, and 50 ppm, based on the Au contents), and the DOX concentrations were about 37% of Au@ZIF-8. The viabilities of MCF-7 cells in different treatments groups were assessed by MTT assay after 24 h of incubation (Fig. 3c). The results indicated the group treated with Au@ZIF-8 only exhibited negligible cytotoxicity without laser irradiation. MCF-7 cells treated with DOX shared similar cytotoxicity in both laser and non-laser treatment due to the absence of photothermal response. The cytotoxic effect of combination group (Au@ZIF-8/DOX + laser irradiation) drastically increased to 82% at the highest concentration of 50 ppm, which was much better than single photothermal or chemotherapy group. On the other hand, the synergistic anti-cancer effect in combination group depends on concentration of Au@ZIF-8/DOX. The treatment efficacy was further evaluated by live/dead staining method using Calcein-AM/propidium iodide (PI) dye after 4 h treatment. Without dead cells were observed in PBS- and DOX-treated cells, and all cells display green fluorescence with well-spread morphology (Fig. 3d). In contrast, obvious cell death regions were observed in Au@ZIF-8 treated and Au@ZIF-8/DOX-treated cells, as evidenced by the increased red spots, suggesting the promising potential of photothermal therapy of Au@ZIF-8. Besides, no remarkable necrotic cell was observed for cells exposed to NIR laser alone, indicating the inappreciable side effect of applied laser. Taken together, those results demonstrated that Au@ZIF-8/DOX can realize superior anti-cancer effect under NIR laser irradiation, which was possibly benefited from the well-coordinated chemo-photothermal therapy.

### In vivo chemo-photothermal therapy

**Fig. 4:**
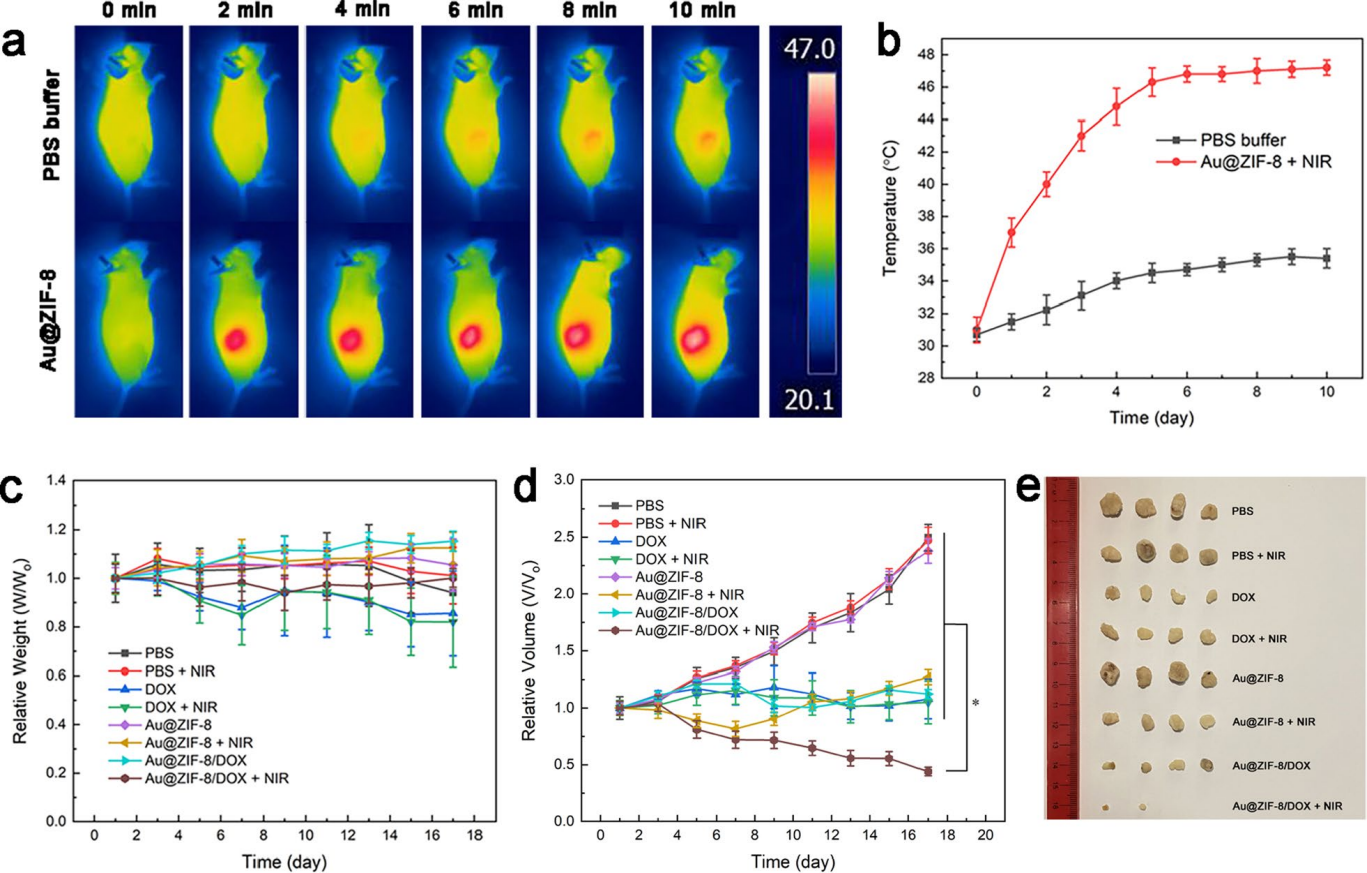
The in vivo chemo-photothermal therapy. **a** in vivo infrared thermal images of MCF-7 tumour-bearing mice after intravenous injection of Au@ZIF-8 or PBS buffer activated by 1 W/cm2 808 nm laser for 10 min. **b** The temperature evaluation profile of tumour site of treated mice under laser irradiation. **c** The relative body weight of tumour-bearing mice in each group during the treatment periods. **d** The relative tumour volumes of mice in each group. **e** The representative photograph of excised tumours from treated mice in each group. Data was represented as mean ± SD (n = 4), *P < 0.05, versus PBS-treated controls

MCF-7 tumour-bearing nude mice were used to evaluate the in vivo therapeutic efficacy of Au@ZIF-8/DOX nanocomplex. The nanoparticle suspension (in PBS) was intravenously injected into the mice prior to perform the photothermal treatment. The in vivo photothermal effect of Au@ZIF-8/DOX nanocomplex was visualized by thermal imaging system (Fig. 4a). The temperature of intratumor area of mouse treated with Au@ZIF-8/DOX nanocomplex increased rapidly to 47 ℃ under the laser irradiation (Fig. 4b), which suggesting the efficient accumulation of nanoparticles in tumour site. The enhanced accumulation may be ascribed to the passive targeting phenomenon, governed by enhanced permeability and retention effect (EPR). In contrast, the negative control group, with mice treated with PBS buffer only, demonstrated limited temperature increment at tumour site. Then, the tumour inhibition rate of Au@ZIF-8/DOX nanocomplex was systematically evaluated. The body weight of mice in each group were recorded in every two days within 17 days. As shown in Fig. 4c, mice treated with PBS (with or without NIR laser) exhibit gradually growing body weight in the first 13 days, followed by weight stable in the rest of days. Mice treated with DOX gradually loss weight in the late stages of the treatment due to the side effect of chemotherapy. For mice treated with Au@ZIF-8/DOX and laser irradiation, the body weight of mice decreased slightly in the first half time and gradually recovered to its original state by the second half time. These results indicated that Au@ZIF-8, as drug carrier for DOX, can efficiently reduce the toxicity of DOX and potentially, exerting the therapeutic effects of DOX in a controlled manner.

To acquire more insights in cancer treatment, the tumour volume of all mice has been calculated and recorded throughout the assessment time. As shown in Fig. 4d, e, tumours were grown aggressively within 17 days without the intervention of DOX and PTT respectively (groups: PBS, PBS + NIR, and Au@ZIF-8). In contrast, the tumour volume in groups (DOX, DOX + NIR, and Au@ZIF-8/DOX) were strictly restrained in their original state. Importantly, the combination group (Au@ZIF-8/DOX + laser irradiation) exhibited best inhibition effect on tumour growth than other experimental groups. It was encouraging that half of tumour were completely eliminated in the combination groups. Although the photothermal therapy alone (Au@ZIF-8 + laser irradiation) also can reduce the tumour growth at the beginning of treatment, the tumour relapse and continuous to growth in the following days. Comparatively, the tumor growth of combination group was completely inhibited during the studied period. On the basis of those results, it can be concluded that Au@ZIF-8/DOX-based synergistic treatment offers more efficient approach than the single-modal therapy.

### The cytotoxicity evaluation of Au@ZIF-8 nanocomplexes

The intracellular cytotoxicity of Au@ZIF-8 nanocomplex at diverse concentrations were studied on cancer cell lines and normal cell lines, including HeLa (cervical cancer cell line), MCF-7 (breast cancer cell line) cell, NIH3T3 cell (normal embryonic fibroblast cell line), and HUVECs cells (human umbilical vein endothelial cells). After co-incubating 24 h, the cell survival rates were all over 90% even at high concentration (up to 400 ppm) of Au@ZIF-8 (Additional file 1: Fig. S5), clearly indicating the excellent biocompatibility of Au@ZIF-8. Such negligible cytotoxicity could be attributed to the encapsulation of PEG2000PE polymer, which has been well recognized as modification materials for improving the hydrophily and biocompatibility of nanoparticle [34]. The haemolysis analysis on Au@ZIF-8 was further conducted to evaluate the hemocompatibility in blood samples. Briefly, a wide range of concentration of Au@ZIF-8 nanoparticles, which covering from 0–200 ppm, was incubated with diluted RBC suspension (in PBS) for 4 h. DI water and PBS buffer were taken as positive and negative controls. The typical absorption peak of haemoglobin at 541 nm was not found for the samples at various concentrations of Au@ZIF-8 when compared with positive control. The haemolytic percentage of Au@ZIF-8 was quantitatively analysed based on the absorbance value. The haemolysis percentages of Au@ZIF-8 were all less than 4% in the pre-designed concentrations (Additional file 1: Fig. 5a), suggesting the negligible haemolytic activity of Au@ZIF-8, which would benefit to in vivo long-term circulation in blood.

**Fig.5:**
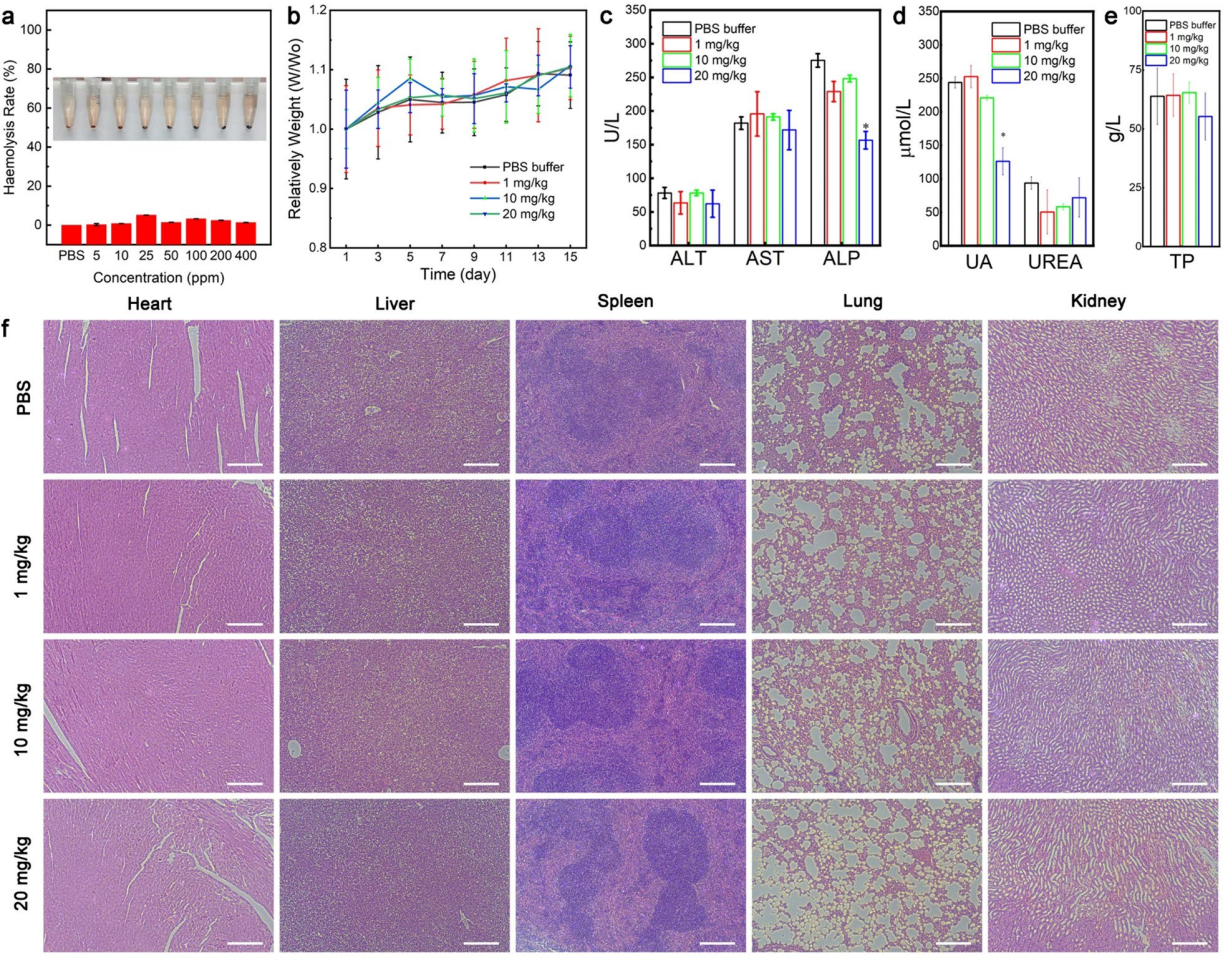
In vitro and in vivo toxicity evaluation of Au@ZIF-8 nanoparticles. **a** The haemolysis result of Au@ZIF-8 with different concentrations. **b** The body weight changes of mice with different treatments over 15 days. The blood biochemistry analysis for **c** liver functions, **d** kidney functions and **e** blood fat levels at day 15 after treatments. uric acid, UA; blood urea nitrogen, UREA; alanine transaminase, ALT; aspartate transaminase, AST; alkaline phosphatase, ALP; total protein, TP; triglyceride. **f** Haematoxylin and eosin (H&E) staining images of major organs (heart, liver, spleen, lung, and kidney) of mice treated with different doses of Au@ZIF-8 nanoparticles. Data was represented as mean ± SD (n = 4). Scar bar: 200 μm

### Biocompatible assessment of Au@ZIF-8 nanocomplexes

Furthermore, the in vivo biosafety of Au@ZIF-8 nanocomplex were systematically evaluated by blood analysis and histological evaluation. The BALB/c mice were randomly divided into four groups and intravenously injected with PBS and Au@ZIF-8 of three dose schemes (1, 10, 20 mg·kg^−1^). Overall, all groups of mice exhibited normal change profile on body weight with slightly increase within 17 days (Fig. 5a). The blood typical blood routine index and biochemical indicators of all treated mice were maintained within the normal range (Fig. 5b, e). Especially, kidney/liver indexes of Au@ZIF-8 treated groups displayed no abnormality as compared to control group, thereby suggesting that the prepared Au@ZIF-8 do not induce deleterious effects. In general, the liver and kidney were the mainly affected organs involved in nanotoxicology due to the nonspecific aggregation. The biocompatibility of Au@ZIF-8 may be due to the rapid clearance effect of reticulo-endothelial system. Meanwhile, it can reasonably conclude that the biodegradability of Au@ZIF-8 would also benefit to its elimination from organs, thus reducing the risk of long-term retention. Further, The H&E-stained tissue section indicated that the major organs (heart, liver, spleen, lung, and kidney) of Au@ZIF-8 nanoparticles treated mice have normal cell morphology and no abnormal pathological changes even at the highest dosage of 20 mg·kg^−1^ (Fig. 5f). These results well implied the good biocompatibility and negligible toxicity of Au@ZIF-8 in vivo, indicating the promising potential for future clinical translation.

## Conclusions

In summary, a novel design of a multifunctional nanoplatform, composed of AuNR, ZIF-8 and DOX, was successfully fabricated via facile method. Attributed to the unique porous structure of ZIF-8 shell, a remarkable DOX loading efficiency of ~ 37% were achieved. Under weak acidic condition and photothermal effects, a controlled drug release phenomenon was observed, which gave rise to a multiple stimuli-responsive cancer therapy. Under the irradiation of 808 nm laser, highly efficient chemo-photothermal therapy was realized both in vitro and in vivo experiments. Moreover, the high biocompatibility of Au@ZIF-8 was confirmed by in vivo study. Considering the synergistic therapeutic effect and excellent biosafety, Au@ZIF-8 has great potential as a promising nanoplatform for cancer therapy in future.

## Supplementary Information

**Additional file 1: Figure S1.** Images of the aqueous solutions for DOX, Au@ZIF-8 and Au@ZIF-8/DOX respectively, under natural day light. ** Figure S2.** Standard curve of DOX in different concentrations.** Table S1.** Drug loading content and loading efficiency of Au@ZIF-8/DOX in different weight ratios.** Figure S3.** TEM images for decomposed Au@ZIF-8 (scale bar: 200 nm) (**a**) after 808 nm laser irradiation in 10 minutes, (**b**) in Ph=5.8 weak acidic aqueous condition for 24 hours.** Figure S4.** The confocal images of MCF-7 cells after incubated with Au@ZIF-8/DOX for 4 hours.** Figure S5.** Cytotoxic evaluation of Au@ZIF-8 on HeLa (cervical cancer cell line), MCF-7 (breast cancer cell line) cell, NIH3T3 cell (normal embryonic fibroblast cell line), and HUVECs cells (human umbilical vein endothelial cells).

## Data Availability

All data generated or analysed during this study are included in this published article.
