# Supplementary material for: Metal organic framework-coated gold nanorod as an on-demand drug delivery platform for chemo-photothermal cancer therapy
Source: J Nanobiotechnology. 2021 Jul 19;19:219. doi: 10.1186/s12951-021-00961-x (PMC8287674; doi:10.1186/s12951-021-00961-x)
Supplement: Supplementary file 1 — Additional file 1: Figure S1. Images of the aqueous solutions for DOX, Au@ZIF-8 and Au@ZIF-8/DOX respectively, under natural day light. Figure S2. Standard curve of DOX in different concentrations. Table S1. Drug loading content and loading efficiency of Au@ZIF-8/DOX in different weight ratios. Figure S3. TEM images for decomposed Au@ZIF-8 (scale bar: 200 nm) (a) after 808 nm laser irradiation in 10 minutes, (b) in Ph=5.8 weak acidic aqueous condition for 24 hours. Figure S4. The confocal images of MCF-7 cells after incubated with Au@ZIF-8/DOX for 4 hours. Figure S5. Cytotoxic evaluation of Au@ZIF-8 on HeLa (cervical cancer cell line), MCF-7 (breast cancer cell line) cell, NIH3T3 cell (normal embryonic fibroblast cell line), and HUVECs cells (human umbilical vein endothelial cells). [file 12951_2021_961_MOESM1_ESM.docx]

**Supporting Information**

**Metal Organic Framework-Coated Gold Nanorod as an On-demand Drug Delivery Platform for Chemo-Photothermal Cancer Therapy**

Junfeng Huang^1,2, #^, Zhourui Xu^3,#^, Yihang jiang^3^, Wing-cheung Law^4^, Biqin Dong^5^, Xierong Zeng^1^, Mingze Ma^3^, Gaixia Xu^3,^ ^*^, Jizhao Zou^1, *^, Chengbin Yang^3, *^

^1^Shenzhen Key Laboratory of Special Functional Materials & Shenzhen Engineering Laboratory for Advance Technology of ceramics, College of Materials Science and Engineering, Shenzhen University, Shenzhen, 518060, China

^2^Key Laboratory of Optoelectronic Devices and Systems of Ministry of Education and Guangdong Province, College of Physics and Optoelectronic Engineering, Shenzhen University, Shenzhen 518060, China

^3^Guangdong Key Laboratory for Biomedical Measurements and Ultrasound Imaging, School of Biomedical Engineering, Shenzhen University Health Science Center, Shenzhen, 518060, China

^4^Department of Industrial and Systems Engineering, The Hong Kong Polytechnic University, Hong Kong 999077, China

^5^Guangdong Provincial Key Laboratory of Durability for Marine Civil Engineering, College of Civil and Transportation Engineering, Shenzhen University, Shenzhen 518060, China.

* Correspondence: [cbyang@szu.edu.cn](mailto:cbyang@szu.edu.cn); [xugaixia@szu.edu.cn](mailto:xugaixia@szu.edu.cn); [zoujizhao@szu.edu.cn](mailto:zoujizhao@szu.edu.cn)

^#^ Junfeng Huang and Zhourui Xu contributed equally to this work.


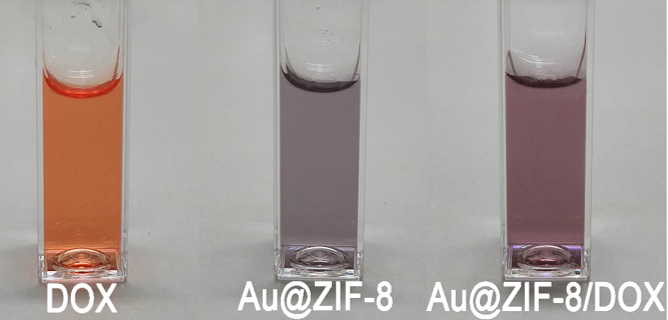


**Figure S1.** Images of the aqueous solutions for DOX, Au@ZIF-8 and Au@ZIF-8/DOX respectively, under natural day light.


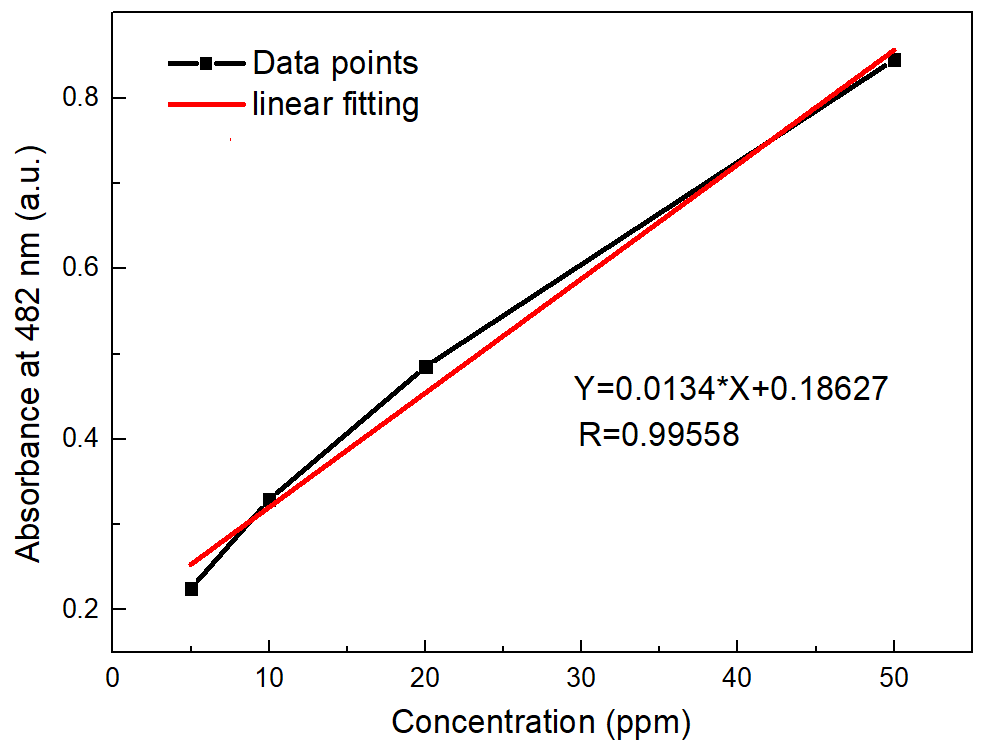


**Figure S2.** Standard curve of DOX in different concentrations.

**Table S1.** Drug loading content and loading efficiency of Au@ZIF-8/DOX in different weight ratios.

| Molar ratio^1^ | Drug loading content (%) | Drug loading efficiency (%) |
| --- | --- | --- |
| 1:1 | 32.12 | 92.861 |
| 2:1 | 36.24 | 91.053 |
| 3:1 | 37.32 | 87.918 |
| 4:1 | 36.78 | 85.273 |
| 5:1 | 36.82 | 84.033 |

^1^ molar ratio between Au@ZIF-8 and DOX


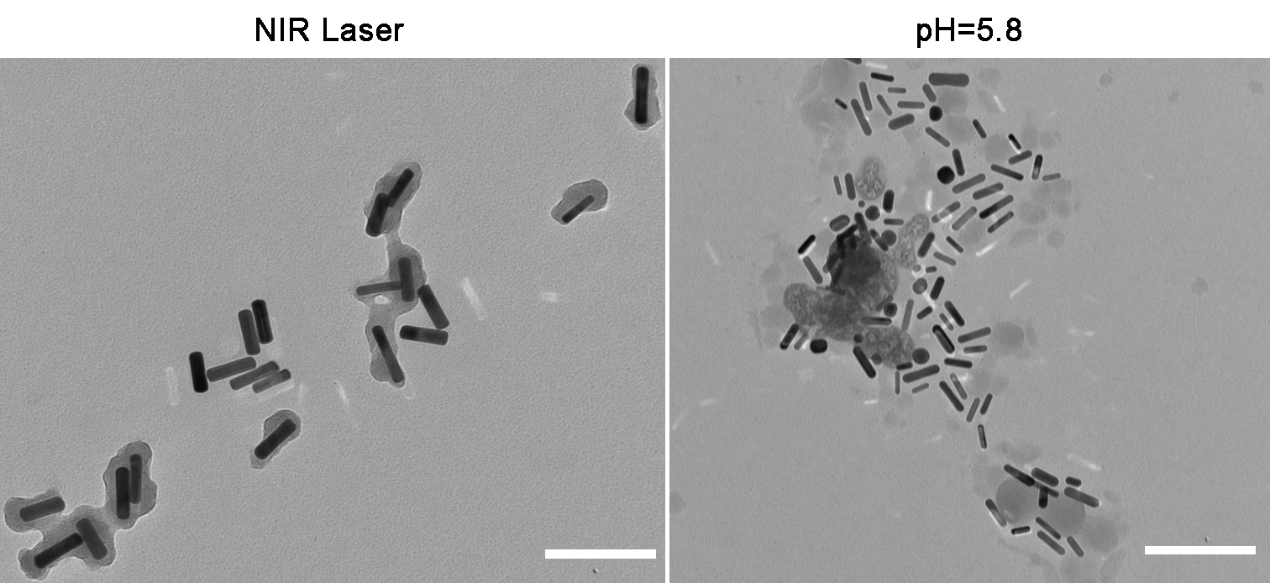


**Figure S3.** TEM images for decomposed Au@ZIF-8 (scale bar: 200 nm) (a) after 808 nm laser irradiation in 10 minutes, (b) in Ph=5.8 weak acidic aqueous condition for 24 hours.


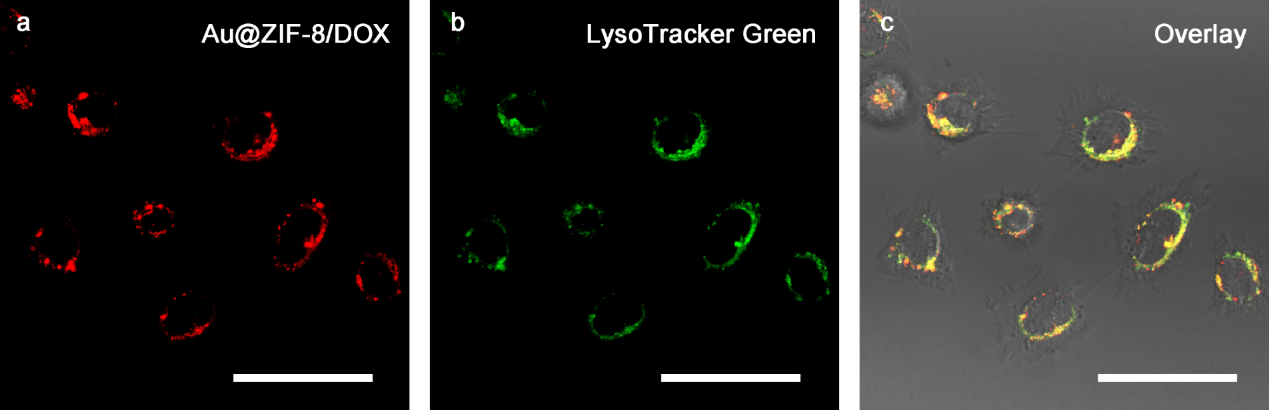


**Figure S4.** The confocal images of MCF-7 cells after incubated with **Au@ZIF-8/DOX** for 4 hours. (a) The red fluorescence of DOX indicates the locations of **Au@ZIF-8/DOX**. (b) The green fluorescence from LysoTracker Green indicates the location and amounts of lysosome inside cells. (c) The overlay image implies an endocytosis process of MCF-7 cells. (Scale bar=25 μm)





**Figure S5.** Cytotoxic evaluation of Au@ZIF-8 on HeLa (cervical cancer cell line), MCF-7 (breast cancer cell line) cell, NIH3T3 cell (normal embryonic fibroblast cell line), and HUVECs cells (human umbilical vein endothelial cells). After cells were incubated with different concentrations of Au@ZIF-8 for 24 hours, the cell viability was analyzed by MTT assay.
